# Endobronchial cryptococcosis in a patient with an HIV infection

**DOI:** 10.1590/0037-8682-0579-2022

**Published:** 2023-02-20

**Authors:** Eduardo Felipe Barbosa Silva

**Affiliations:** 1 Hospital de Base do Distrito Federal, Unidade de Broncoscopia, Brasília, DF, Brasil.

**FIGURE 1:**
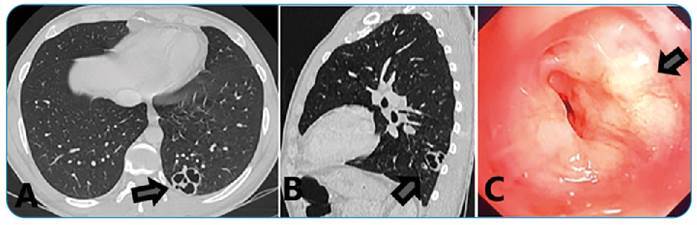
**(A,B)** "Excavated and septate lobulated formation in the left lower lobe. Lymph node enlargement in the left pulmonary hilum." **(C)** Infiltrative lesion in left lower lobe bronchus (superior segment).

A 30-year-old man with a diagnosis of AIDS discontinued anti-retro viral therapy one year prior to presentation for a headache, fever and weight loss evaluation. Chest computed tomography revealed excavated and septate lobulated formation in the left lower lobe (Figure 1A-B ), and bronchoscopy revealed an infiltrative lesion in the left lower lobe bronchus (superior segment)-(Figure 1C). The bronchial biopsy specimens showed many small-sized non-budding yeast-like structures, with periodic acid-Schiff staining positivity, compatible with *Cryptococcus neoformans*. *Cryptococcus neoformans* was also cultured from both blood and cerebrospinal fluid. The patient was treated with intravenous liposomal amphotericin B and fluconazole and underwent cerebrospinal fluid drainage. He followed an uncomplicated disease course.

Cryptococcosis preferentially affects immunocompromised hosts (patients with HIV infection, those undergoing transplantation, those using high-dose corticosteroids, and those with diabetes mellitus, chronic renal failure, or other such diseases^1^. Although the lungs serve as a gateway to this infection, extrapulmonary forms (for example meningitis) represent the most common clinical presentations^1^ ^,^ ^2^. Most patients initially show nonspecific respiratory clinical manifestations^2^. Radiological findings include parenchymal infiltrates, cavitated lesions, lymphadenopathy, pleural effusion, and pulmonary masses and nodules^1^ ^,^ ^3^. Endobronchial cryptococcosis is a rare manifestation of pulmonary infection and endoscopically presents as a vegetating, polypoid, plaque-like lesion, submucosal infiltration, or an ulcer^1^ ^,^ ^3^. In summary, *Cryptococcosi*s should be considered in the differential diagnosis of endobronchial lesions in immunocompromised patients, especially in those with AIDS.
